# Fully automated measurement system for temperature-dependent X-ray total scattering at beamline BL04B2 at SPring-8

**DOI:** 10.1107/S1600577521013527

**Published:** 2022-01-18

**Authors:** Hiroki Yamada, Kengo Nakada, Michitaka Takemoto, Koji Ohara

**Affiliations:** a Japan Synchrotron Radiation Research Institute (JASRI), 1-1-1 Kouto, Sayo-cho, Sayo-gun, Hyogo 679-5198, Japan

**Keywords:** X-ray scattering, automation, synchrotron beamline, pair distribution functions, temperature-dependence

## Abstract

Data-driven approaches in materials science demand the collection of large amounts of data on the target materials at synchrotron beamlines. To accurately gather suitable experimental data, it is essential to establish fully automated measurement systems to reduce the workload of the beamline staff. Moreover, the recent COVID-19 pandemic has further emphasized the necessity of automated and/or remote measurements at synchrotron beamlines. Here, the installation of a new sample changer combined with a high-temperature furnace and a fully automated alignment system on beamline BL04B2 at SPring-8 is reported. The system allows X-ray total scattering measurements of up to 21 samples at different temperatures (from room temperature to 1200°C) to be conducted without any human assistance.

## Abstract 2

The implementation of a new, fully automated X-ray total scattering system on beamline BL04B2 at SPring-8 is described.

## 1. Introduction

Data-driven approaches in materials science have attracted significant attention (Spurgeon *et al.*, 2021 ▸) for understanding properties that cannot be predicted by more conventional methods. For example, the properties of high-entropy alloys were revealed from a combination of experimental and simulated data using an informatics approach (Rickman *et al.*, 2019 ▸). The more data available for analysis, the better the performance of these approaches. Therefore, maximizing the amount of data collected about materials is beneficial. To accurately gather such vast amounts of experimental data, it is essential to establish fully automated measurement systems due to limitations with and/or adverse effects from human resources.

Many synchrotron beamlines for powder X-ray diffraction (XRD), small-angle X-ray scattering (SAXS) and X-ray absorption spectroscopy (XAS) [*e.g.* BL02B2 in SPring-8 (Kawaguchi *et al.*, 2017 ▸), BL14B2 in SPring-8 (Oji *et al.*, 2012 ▸), BL19B2 in SPring-8 (Osaka *et al.*, 2016 ▸), ROBL-II in ESRF (Figueroa *et al.*, 2018 ▸), B18 in Diamond Light Source (Figueroa *et al.*, 2018 ▸) and the P12 beamline in PETRA III (Blanchet *et al.*, 2015 ▸)] have already developed automated measurement systems that collect significant quantities of data. The automation not only improves the accuracy of measurements to reduce human error but also benefits the new users who lack familiarity with synchrotron radiation experiments.

Looking at the beamlines dedicated to X-ray pair distribution function (PDF) analysis, advanced beamlines such as P02.1 at PETRA III (Dippel *et al.*, 2015 ▸), ID-22 at ESRF (Fitch, 2008 ▸), 11-ID-B at APS (Chupas *et al.*, 2007 ▸) and 28-ID at NSLS II (Palomino *et al.*, 2017 ▸) use automated sample changers for high-throughput measurements. For example, in P02.1 at PETRA III, the automatic sample-exchange robot, which can treat up to 300 samples, is available for measurements under ambient conditions. However, there was previously no beamline available for the fully automated measurement of X-ray total scattering for PDF analysis in Japan. A fully automated measurement system for this analysis method is highly desired at SPring-8.

Moreover, the COVID-19 pandemic has made it difficult for researchers to travel to conduct experiments at foreign synchrotron facilities. Many experiments for overseas users were canceled during the past year at our facility, undeniably delaying the progress of scientific research. Sending samples directly to the synchrotron facilities has become the only way to obtain highly sophisticated synchrotron experimental data for most scientists. Automated measurements in the synchrotron beamlines can be monitored and controlled remotely, removing the necessity of physically traveling to the beamline site.

We installed a new sample changer combined with a high-temperature furnace and fully automated alignment system on beamline BL04B2 at SPring-8. The system allows X-ray scattering measurements of up to 21 samples at different temperatures to be performed automatically. Examples of typical measurements of X-ray total scattering and PDF analyses are also discussed to show the validity and usefulness of this system.

## 2. Experimental

### 2.1. Hardware setup

The light source on BL04B2 is a bending magnet with single bent-crystal monochromators which provide a horizontally focused beam with maximized flux at the target. X-rays with energies of 37.8 keV (Si111), 113 keV (Si333) and 61.3 keV (Si220) are available at this beamline as the angle of the monochromator is fixed to 3°. By adjusting the bending radius of the monochromators between 320 m and 430 m, the X-rayfocal position in the experimental systems could be correspondingly tuned between 10 m and 15 m from the monochromator according to the experimental demands (Isshiki *et al.*, 2001 ▸). The distance between the monochromator and the center of the diffractometer is 10 m and the typical size of the beam at the sample is 2.0 mm (V) × 2.0 mm (H).

**Figure 1:**
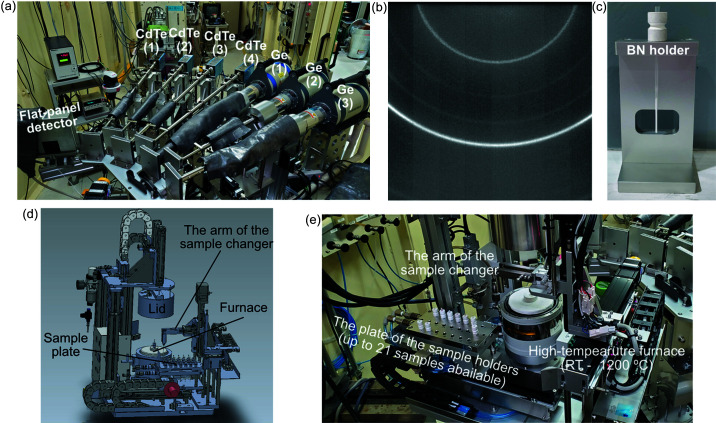
Experimental setup of the horizontal diffractometer and automated sample changer with a furnace in BL04B2. (*a*) Seven semiconductor detectors, and the flat-panel detector fixed on the diffractometer at around −15.3°. (*b*) Typical two-dimensional XRD pattern captured by the flat-panel detector. (*c*) Boron nitride holder containing a quartz capillary (with a diameter of 1.5 mm) and the aperture used to check the height of the sample. (*d*) Automated sample changer and furnace. Up to 21 samples can be loaded on the plate. (*e*) Photograph of the automated sample changer on the diffractometer.

No double-crystal monochromators or X-ray mirrors are incorporated in the BL04B2 optics. This is useful for PDF analyses that require high scattering intensities in the high-*Q* region, but also means that X-ray harmonics are directly injected into the experimental system. Therefore, it is essential to use detectors with energy-resolution abilities to discriminate the higher harmonics; hence, seven semiconductor detectors [four CdTe detectors (X-123CdTe, Amptek, USA) and three Ge detectors (GL-0515R, Cambella, USA)] are positioned at 8° intervals on the horizontal diffractometer [Fig. 1 ▸(*a*)]. Moreover, to minimize the background scattering from the surroundings, the flight path with a double-slits system is prepared prior to each measurement. Detailed features of these detectors and the experimental setup have been discussed previously (Ohara *et al.*, 2020 ▸). The signals from the detectors are treated by either the signal processors provided by the manufacturers of the detector or a digital signal processor (APN504XGbE-L, TechnoAP, Japan). The signal from the region of interest is collected by a 16-channel counter (CT16-01F, Tsuji Electronics, Japan). The scattering data obtained from the detectors can be combined using an established procedure (Kohara *et al.*, 2007 ▸). We also used a two-dimensional flat-panel detector (C10013SK, Hamamatsu Photonics, Japan) to check the sample quality and Bragg angle and to determine the suitable receiving slit size for the semiconductor detectors [a typical image is shown in Fig. 1 ▸(*b*)]. This flat-panel detector (FPD) is fixed on the diffractometer at around −15.3° from the first detector to avoid interference with the measurement [Fig. 1 ▸(*a*)].

The new automated sample changer system (manufactured by Rigaku Aihara Seiki, Japan) can load up to 21 samples [Figs. 1 ▸(*c*) and 1(*d*)]. Capillaries containing the samples of interest are placed in the boron nitride (BN) holders and held by the sample plate. The BN holders accept capillaries with diameters of 1.5 mm or 2.0 mm [Fig. 1 ▸(*c*)]. Prior to measurement, the sample is inserted into the furnace (HT1500, high-temperature attachment, Rigaku, Japan) from the sample plate and sealed under vacuum. The sample in the furnace is heated by the thermal contact and radiation (especially at high temperature) and the temperature is calibrated using a thermocouple. The heating rate of this furnace is set at 10°C min^−1^ as the default and the maximum rate is 20°C min^−1^ if the sample does not move due to the rapid heating. Automated high-energy XRD and X-ray total scattering temperature-dependent measurements (from room temperature to 1200°C) are also available on the same system.

### 2.2. Automated X-ray total scattering procedure

**Figure 2:**
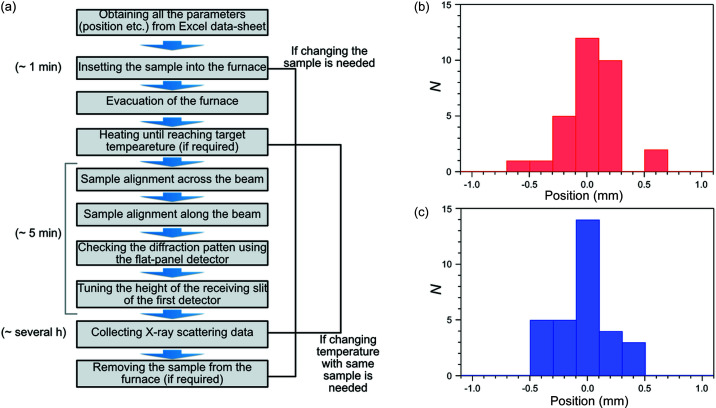
Automated X-ray total scattering measurement procedure and reproducability of the sample position. (*a*) Automated X-ray scattering measurement procedure. This cycle is automatically repeated until all the measurement conditions have been completed. The typical time required for each step is also shown. (*b*) Histogram of the sample position across the beam. (*c*) Histogram of the sample position along the beam. Note that *N* represents the number of appearances during the reproducability test.

The fully automated measurement system in BL04B2 is achieved by combination of the hardware and software developments described above. Details of the operation procedure are summarized in Fig. 2 ▸.

First, the sample capillary in its BN holder is inserted into the furnace. The furnace is evacuated to suppress scattering from air around the sample. If heating is required, the temperature is raised until the target temperature is reached. After waiting for the temperature to stabilize, the sample position across the beam is determined by the X-ray absorption approach. The intensity of the penetrated X-rays through the sample becomes lowest when the sample is on the center and thus moving to that position ensures that the sample is on the center of the beam. After the sample is moved, the position along the beam is determined by the first semiconductor detector, shown as CdTe(1) in Fig. 1 ▸(*a*), with double slits at a higher angle using scattered X-rays. Because of the limited light-path made by the double-slits between the detector and the sample, scattered intensity becomes highest when the sample is on the center of the diffractometer, so that the position along the beam can be obtained from the intensity profiles. The sample changer is mounted on an independent electric stage; thus, no interference occurs between the sample alignment and the movement of the sample changer. After correctly aligning the sample, the scattering pattern at low angles (up to around 8°) is obtained using the flat-panel area detector to check the Bragg angle and save the two-dimensional diffraction image in order to confirm the quality of the sample later. If no scattering pattern can be obtained, the sample is considered unacceptable and the automated system proceeds to measure the next sample.

Further analysis of the X-ray scattering data around the angle with highest intensity using the first detector is conducted to avoid saturation. The scattering intensity is tuned by adjusting the receiving slits prior to the detectors. Typically, the alignment process takes around 5 min which is comparable with the time taken when manually conducted by the beamline staff. Finally, measurements of the high-energy XRD and/or X-ray total scattering are conducted. The data obtained were saved as a file containing the scattering intensities and incident X-ray flux from each detector with the angles, which can be reduced to the single diffraction profile and the PDF in the provided software at BL04B2.

After measurement under one condition, the program checks the input sheet to determine whether a change of sample is required for the next measurement. If the answer is yes, the previous sample is automatically removed from the furnace and the next sample is inserted by the sample changer. If the answer is no, the temperature is adjusted until the new target temperature is reached. By continuing this sequence until the end of the measurement condition input sheet is reached, all the data are obtained automatically with no further input.

Note that no specific technical experience is required to operate this system and all the parameters for the measurements can be loaded from an *Excel* datasheet. Therefore, even researchers who are not experts in synchrotron experiments or X-ray total scattering measurements can easily use this fully automated measuring system.

## 3. Results and discussion

To check the validity of this measurement system, we checked the reproducibility of the sample position adjustment 30 times with the same sample capillary. From the statistical analysis, we determined that the standard deviations of the positions across and along the beam were 0.21 mm and 0.24 mm, respectively [Figs. 2 ▸(*b*) and 2(*c*)]. These values are considered reasonable because the play between the BN holder and the stage in the furnace is around 0.2 mm and indicate that the standard deviations of the sample positions both across and along the beam are small enough compared with the tunable range of the motors and the size of the X-rays at the sample position. Thus the alignment range of this system is suitable in this beamline. To determine the required accuracy for setting the sample at the center of the diffractometer, Rietveld analyses of NIST CeO_2_ powder diffraction patterns (packed in a capillary with a diameter of 1 mm) were conducted (Petříček *et al.*, 2014 ▸). The sample positions 0.15 mm and 0.3 mm from the center (the direction across the beam) were compared. The energy of the X-rays was 112.58 keV and the scattering angle was measured at steps of 0.0025° between 0.3° and 9.0°. *R*_wp_ values of the refinements are 9.3%, 10.6% and 12.7%, respectively. From these results, the scanning step to move the sample at the center was determined to be 0.05 mm. Since BL04B2 is mainly dedicated to PDF analysis, it does not employ mirrors and has an intensity-oriented optical system with a single monochromator. In other words, the experimental setup is not for the higher-resolution measurement but for the measurement with higher scattering intensity with lower background. This is the reason for a relatively higher value of *R*_wp_ compared with other beamlines dedicated to high-resolution X-ray diffraction.

In addition, we conducted X-ray total scattering experiments on silicate glass at room temperature and on zeolite at different temperatures. The X-ray energy used in these measurements was 61.27 keV and data were obtained in the scattering angle range between 0.3° and 48°. The maximum *Q* (*Q*_max_, where *Q* = 4πsinθ/λ) observed was 26 Å^−1^ for silicate glass and 20 Å^−1^ for zeolites. The obtained data were handled by applying established analytical procedures (Kohara *et al.*, 2007 ▸) including absorption, background, polarization and Compton scattering corrections, and were then normalized to obtain the Faber–Ziman total structure factor *S*(*Q*) (Faber & Ziman, 1965 ▸). The *S*(*Q*) values obtained with the window function developed by Lorch (1969 ▸) were used to calculate the reduced PDF, *G*(*r*), according to the following equation (Billinge, 2008 ▸):

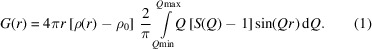

Here *G*(*r*) represents the histogram of the atomic distances inside the materials on an atomic scale; thus by analyzing this profile, one can understand the atomic arrangement even if the material is non-crystalline.

**Figure 3:**
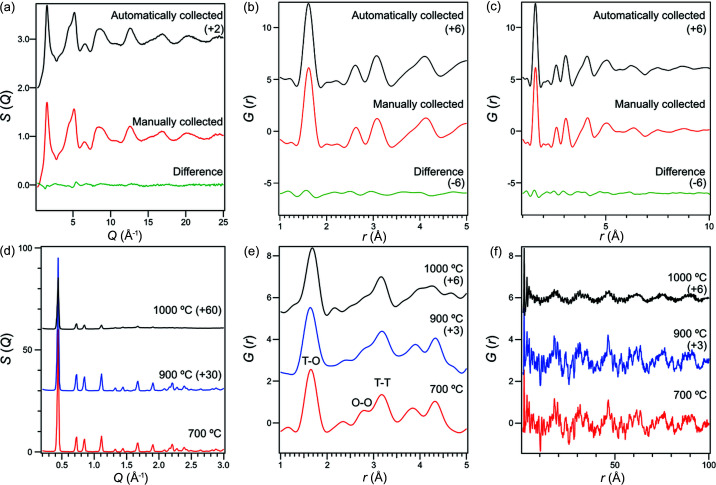
Data obtained from automated X-ray total scattering measurements. (*a*) *S*(*Q*) of silicate glass. (*b*) Comparison of *G*(*r*) values of silicate glass. (*c*) Comparison of *G*(*r*) values of silicate glass in a wide range. (*d*) *S*(*Q*) profiles of FAU zeolites at different temperatures. (*e*) *G*(*r*) values of FAU zeolites at different temperatures. (*f*) *G*(*r*) values of FAU zeolites at different temperatures with a wide range.

Fig. 3 ▸(*a*) shows *S*(*Q*) of the silicate glass obtained using both automatically collected and manually collected data. The value for *S*(*Q*) obtained is consistent with the previous work aligned manually (*R*_p_ value is 1.64%), indicating the validation of this automated system. This result is not surprising when the sample is positioned at the center of the diffractometer by the fully automated system. The *G*(*r*) values calculated from these *S*(*Q*) values are also shown in Figs. 3 ▸(*b*) and 3 ▸(*c*). Because of the truncation error of Fourier transformation, a small difference is observed but is still comparable with the previous profile.

Thermal stability experiments were conducted on faujasite (FAU) zeolites (HSZ-320NAA, Tosoh, Japan) by heating in the furnace. The gradual amorphization of the FAU zeolites is indicated by the intensity change in the *S*(*Q*) patterns as the temperature increases [Fig. 3 ▸(*d*)]. The PDFs of the FAU samples are shown in Figs. 3 ▸(*e*) and 3(*f*). The correlations at 1.6 Å, 2.7 Å and 3.1 Å correspond to the nearest *T*–O, O–O and *T*–*T* pairs, respectively (where *T* represents either an Si or an Al atom). Focusing on O–O correlation, the intensity of this peak becomes smaller at 900°C and 1000°C (see Fig. S1 and Table S1 of the supporting information). This means that structural distortion around O–O is larger than the correlations of *T*–*T*, and might indicate that structural collapse has occurred from the destruction of this O–O structure. The peaks above 3.5 Å can mainly be attributed to the aluminosilicate ring structure. The PDF profiles demonstrate the collapse of the ordered ring structure at around 3.7 Å after heating at 1000°C and the disappearance of the correlation around 4.4 Å. The correlations in the longer-range region [Fig. 3 ▸(*f*)] also demonstrate that the ordered structure derived from the cage of FAU zeolites (around 16 Å) still remains after the degradation at 1000°C, despite of the deformation of the local structure (∼5 Å) proceeding. This tendency is consistent with XRD results which show the remaining Bragg peaks from the zeolite.

Understanding the degradation process is important in the preparation of materials with higher stability (Iyoki *et al.*, 2018 ▸). The method developed in this study facilitates the collection of data for heat-degradation experiments that can be conducted *in situ* and through an automatic process. In addition, the fully automated measurement system is expected to be used not only for observing pyrolysis processes but also for the optimization of material preparation processes by utilizing the measurement system to visualize local structural information. The automation will enhance data-driven science, helping us to understand and realize innovative processes for future industrialization.

## 4. Conclusions

We installed a new sample changer combined with a high-temperature furnace and a fully automated alignment system on beamline BL04B2 at SPring-8. Temperature-dependent X-ray total scattering measurements for up to 21 samples could be performed without any human interaction in the new system, which is a significant upgrade from the previous sample changer for ambient conditions. The XRD and PDF data obtained were fairly consistent with the existing data. The new automated system will support and accelerate optimization of material synthesis processes by enabling the visualization of local structural information during the process and aiding data-driven science to develop innovative processes that will lead to industrial applications. Combination with other important measurement techniques such as synchrotron powder XRD and XAS will benefit the future of cutting-edge science.

## Supplementary Material

Supporting information file. DOI: 10.1107/S1600577521013527/gy5027sup1.pdf
