# Supplementary material for: Fully automated measurement system for temperature-dependent X-ray total scattering at beamline BL04B2 at SPring-8
Source: J Synchrotron Radiat. 2022 Jan 18;29(Pt 2):549–54. doi: 10.1107/S1600577521013527 (PMC8900857; doi:10.1107/S1600577521013527)
Supplement: Supplementary file 1 [file s-29-00549-sup1.pdf]

JOURNAL OF SYNCHROTRON RADIATION

Volume 29 (2022)

Supporting information for article:

Fully automated measurement system for temperature-dependent X-ray total scattering at beamline BL04B2 at Spring-8

Hiroki Yamada, Kengo Nakada, Michitaka Takemoto and Koji Ohara

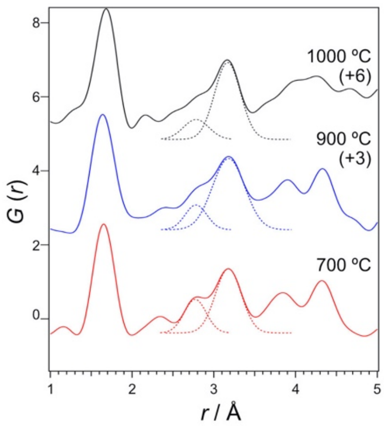

Figure S1. Comparison of *G*(*r*)s of zeolites. The fitted area of O-O and T-T correlations are shown in the figure.

Table S1. Area comparison of *G*(*r*)s of zeolites. The ratio compared to O-O and T-T area are also calculated and summarized.

Temperature	Relative are of O-O	Relative are of T-T	Area ratio (O-O/T-T)
700ºC	0.31	0.69	0.44
900ºC	0.19	0.81	0.23
1000ºC	0.21	0.83	0.25
